# Comparative Transcriptomics of *Flammulina filiformis* Suggests a High CO_2_ Concentration Inhibits Early Pileus Expansion by Decreasing Cell Division Control Pathways

**DOI:** 10.3390/ijms20235923

**Published:** 2019-11-25

**Authors:** Jun-Jie Yan, Zong-Jun Tong, Yuan-Yuan Liu, Yi-Ning Li, Chen Zhao, Irum Mukhtar, Yong-Xin Tao, Bing-Zhi Chen, You-Jin Deng, Bao-Gui Xie

**Affiliations:** 1 Mycological Research Center, College of Life Sciences, Fujian Agriculture and Forestry University, Fuzhou, Fujian 350002, China; junjie017@163.com (J.-J.Y.); ttzjun1@163.com (Z.-J.T.); lyylyy0815@163.com (Y.-Y.L.); liyiining@163.com (Y.-N.L.); 15581771987@163.com (C.Z.); erumm21@yahoo.com (I.M.); taoyongxinmuse@163.com (Y.-X.T.); cbz_2006@163.com (B.-Z.C.); 2 Institute of Edible Fungi, Shanghai Academy of Agricultural Sciences, Shanghai 201403, China; 3 Institute of Oceanography, Minjiang University, Fuzhou, Fujian 350108, China; 4 College of Horticulture, Fujian Agriculture and Forestry University, Fuzhou, Fujian 350002, China; 5 College of Food Science, Fujian Agriculture and Forestry University, Fuzhou, Fujian 350002, China

**Keywords:** winter mushroom, pileus development, carbon dioxide stress, RNA-seq, cell division, ubiquitin–proteasome system, gene expression

## Abstract

Carbon dioxide is commonly used as one of the significant environmental factors to control pileus expansion during mushroom cultivation. However, the pileus expansion mechanism related to CO_2_ is still unknown. In this study, the young fruiting bodies of a popular commercial mushroom *Flammulina filiformis* were cultivated under different CO_2_ concentrations. In comparison to the low CO_2_ concentration (0.05%), the pileus expansion rates were significantly lower under a high CO_2_ concentration (5%). Transcriptome data showed that the up-regulated genes enriched in high CO_2_ concentration treatments mainly associated with metabolism processes indicated that the cell metabolism processes were active under high CO_2_ conditions. However, the gene ontology (GO) categories and Kyoto Encyclopedia of Genes and Genomes (KEGG) pathways associated with cell division processes contained down-regulated genes at both 12 h and 36 h under a high concentration of CO_2_. Transcriptome and qRT-PCR analyses demonstrated that a high CO_2_ concentration had an adverse effect on gene expression of the ubiquitin–proteasome system and cell cycle–yeast pathway, which may decrease the cell division ability and exhibit an inhibitory effect on early pileus expansion. Our research reveals the molecular mechanism of inhibition effects on early pileus expansion by elevated CO_2_, which could provide a theoretical basis for a CO_2_ management strategy in mushroom cultivation.

## 1. Introduction

The pileus is a prominent part in the top of the mushroom fruiting body. It is essential for sexual reproduction and completion of the mushroom life cycle [1]. Pileus expansion often accompanies the formation and ejection of the spores, which leads to the aging of the fruiting-body and the end of the life cycle [2]. For edible mushrooms, pileus expansion leads to the loss of nutrition and the decline of economic value. According to edible mushroom marketing and sales standards, the pileus size and shape are significant agronomic traits and among the most important indexes for product quality [3]. Therefore, it is critical to control pileus expansion during fruiting management of commercially cultivated edible fungus.

Several environmental factors can influence the fruiting body development, including light, temperature, carbon dioxide (CO_2_) concentration, and humidity [2]. However, CO_2_ is one of the most important commonly used environmental factors for controlling pileus expansion during mushroom cultivation. A high concentration (HC) of CO_2_ can significantly inhibit pileus expansion, whereas low concentrations (LC) of CO_2_ could promote the pileus expansion of many mushroom species [4,5,6]. However, the CO_2_ tolerance ability is quite different among different mushroom species and the mechanism of CO_2_ regulation of mushroom development is still unclear, which makes it hard to manage a standard CO_2_ level during mushroom cultivation [7].

Carbon dioxide is an important trace gas in the Earth’s atmosphere. Variation in its concentration can affect the growth and survival of living organisms on the Earth [8,9,10,11]. Previous studies demonstrated that CO_2_ is transported through membranes, sensed by organisms, and acts as a key signaling molecule to control growth, differentiation, virulence, biotic interactions, etc. [12,13,14,15,16,17]. In yeast, CO_2_ can be transported into the cells mainly by simple diffusion, then converted to HCO_3^−^_ and maintains CO_2_/HCO_3^−^_ homeostasis by carbonic anhydrase [18]. CO_2_ can also contribute to morphology, mating, sporulation, phenotypic switching, and virulence processes of fungi via the adenylyl cyclase/cAMP pathway [19]. Recently, Lu et al. (2019) unraveled a new regulatory mechanism of CO_2_ signaling in fungi hyphal development by reducing Ume6 phosphorylation and degradation. However, the knowledge of the CO_2_ regulation mechanism in higher fungi is still unknown [20].

*Flammulina filiformis* (previously known as *F. velutipes*) is an important commercially cultivated mushroom with high dietary and medicinal value. According to recent statistics, its production contributes to more than 10% of the world’s cultivated mushrooms [21,22]. Similar to most of the mushroom’s cultivation methods, the CO_2_ concentration is strictly controlled during the fruiting body development of *F. filiformis*. For mycelial growth and primordia stimulating, a CO_2_ concentration of less than 0.3%–0.5% is required, while a CO_2_ concentration of more than 0.1%–0.2% may support stipe elongation and a decrease in the pileus diameter [23]. On the basis of these findings, maintaining a high CO_2_ level has become one of the main methods to inhibit pileus expansion and is being commonly used in mushroom cultivation factories to produce the high-quality fruiting bodies of *F. filiformis* with a long stipe and small pileus. In fact, the control of the CO_2_ level varies with the period of fruiting body development in mushroom cultivation farms. Practice shows that keeping CO_2_ at a low level during primordium formation and the early stage of fruiting body development and then elevating it to the high-level during pileus expansion, and finally, reducing it before harvesting time is beneficial to gaining high-quality fruiting bodies. Many farms even elevate the CO_2_ levels above 3% to inhibit pileus expansion. However, why a high CO_2_ concentration inhibits pileus expansion and how it works at the molecular level are still unknown, limiting innovation of the mushroom cultivation management strategy.

By the development of sequencing technology, "omics" techniques have been used to study fruiting body morphogenesis [24]. The genomes, transcriptomes, and proteomes of *F. filiformis* strains were published over several years, providing a mass of data to study the mechanism of fruiting-body development [25,26,27,28,29,30,31]. In this study, the growth rhythm of *F. filiformis* pileus was compared under low and high CO_2_ concentrations. A comparative transcriptome analysis was performed to reveal the mechanism of pileus expansion controlled by CO_2_ in *F. filiformis*.

## 2. Results

### 2.1. Phenotype of F. filiformis Fruiting Bodies under Different CO_2_ Concentrations 

**Figure 1:**
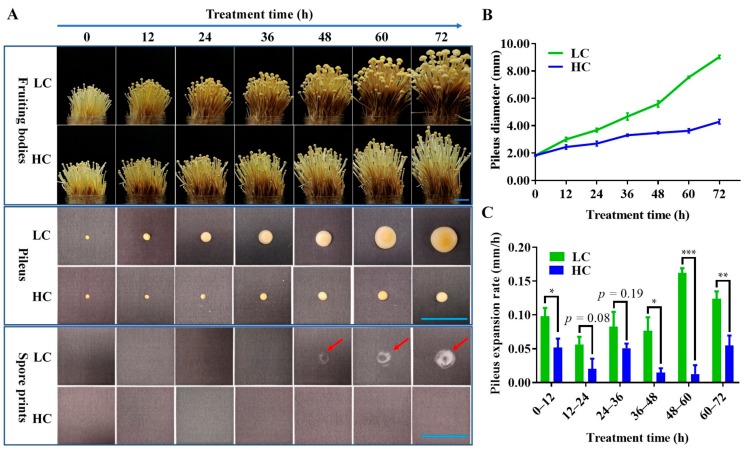
Morphological comparison of fruiting bodies of *Flammulina filiformis* under different CO_2_ concentrations. (**A**) Morphology, pileus size, and basidiospore ejection under low (LC) and high (HC) CO_2_ concentrations. Red arrows indicate the spore sprints. Bar = 2 cm. (**B**) The effects of low and high CO_2_ concentrations on pileus expansion; data are the means of six replicates, and the bars represent SEMs. (**C**) The pileus expansion rates of each 12 h growth time; data are the means of six replicates, and the bars represent SEMs. Significant differences were analyzed using Student’s *t*-tests, * *p* < 0.05, ** *p* < 0.01, *** *p* < 0.001.

**Figure 2:**
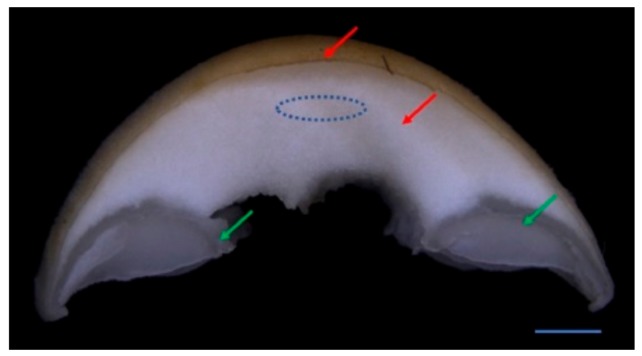
Structural presentation of *F. filiformis* pileus. Red arrows indicate the context, while green arrows indicate lamella. The central region of the context, shown as a dotted line oval, indicates the tissue sampling region for anatomical studies. Bar = 1 mm.

To investigate the effect of in CO_2_ variations on the development of pileus, young fruiting bodies of *F. filiformis* F19 strain (at the stage of a 50 mm stipe length and 2 mm pileus diameter) were kept under a LC or HC of CO_2_. As shown in Figure 1A, the fruiting body development was observed at both low and high CO_2_ conditions; however, the stipes were a little shorter in length, thinner, and the color of fruiting bodies was lighter in the HC group as compared with the LC group. A significant quick pileus expansion and basidiospore ejection at 48 h was recorded in the LC group, whereas the pileus growth was significantly inhibited with no basidiospore shedding in the HC group (Figure 1A). Initially, a difference in the pileus diameter was observed after 12 h of treatment under both LC and HC CO_2_ conditions as compared with the control. A significant (Student’s *t*-test, *p* < 0.05) difference in the pileus diameter was also observed between the LC and HC CO_2_ groups (Figure 1B) from 12 h to 72 h. The expansion rates of the pileus were about 1.9-, 2.8-, 1.6-, 5.2-, 12.8-, and 2.3-fold in the LC group than the HC group during the 0–12 h, 12–24 h, 24–36 h, 36–48 h, 48–60 h, and 60–72 h intervals respectively (Figure 1C). While the pileus growth rate difference was highly significant between the LC and HC groups at 48 h of treatment, abundant basidiospore ejection from the pileus was observed at 48 h in the LC group, suggesting a significant change in the function of the pileus due to the variation in CO_2_ conditions. To investigate the mechanism of the involvement of CO_2_ in pileus expansion, the lamella parts were removed and the context parts of the pileus (as shown in Figure 2) with 12 h and 36 h intervals of CO_2_ treatment were collected for transcriptome analysis.

### 2.2. RNA-Seq Data Analysis of F. filiformis Grown under Different CO_2_ Conditions

**Figure 3:**
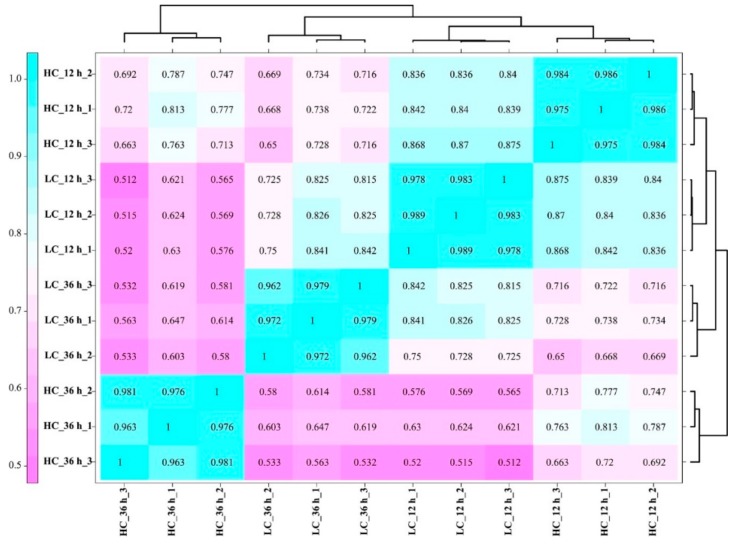
Pearson correlation coefficients for pair-wise comparisons of transcriptome data. The sample names were combined with treatment_time_repeat. HC means high CO_2_ concentration treatment; LC means low CO_2_ concentration treatment.

**Figure 4:**
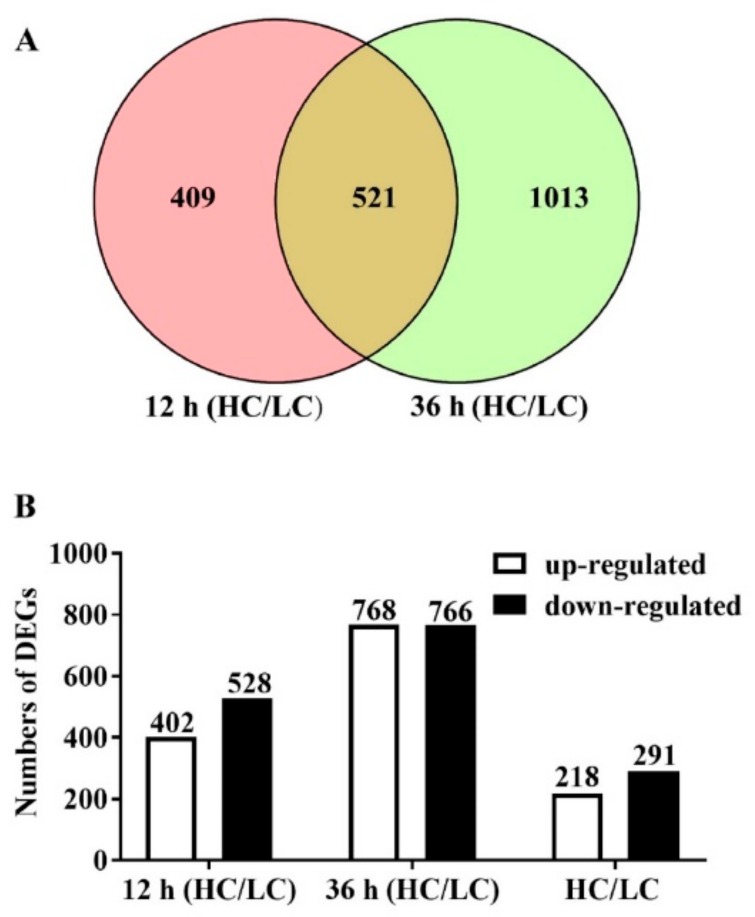
Number of differentially expressed genes (DEGs) in response to different CO_2_ concentrations. (**A**) Venn diagram showing the number of DEGs between high and low CO_2_ concentrations. (**B**) The number of up or down regulated genes following the high CO_2_ treatment compared with the low CO_2_ treatment. The number 12 (HC/LC) corresponds to the number of DEGs after 12 h of treatment, and 36 (HC/LC) corresponds to the number of DEGs after 36 h of treatment, HC/LC corresponds to the DEGs with the same expression patterns after both 12 h and 36 h of treatment. The number of DEGs is indicated on the top of the histograms.

A total of 207.1 million raw reads were generated from 12 samples using Illumina paired-end sequencing. About 203.7 million clean reads were obtained, and the percentage of Q30 bases in each sample was over 91% (Table S1). More than 81% of the total reads mapped to the *F. filiformis* L11 genome for each sample. These results confirmed the reliability of the RNA-seq analysis and the sampling accuracy of the context tissues (the pileus without lamella) used in this study. The gene fragments per kilobase million (FPKM) values were obtained after the mapping of clean reads against the *F. filiformis* genome, and 10726 genes were found to be expressed in at least one sample (Table S2). The relationships between the 12 transcriptomes from four different treatments were analyzed. As shown in Figure 3, the Pearson correlation coefficient of biological repetition samples were about 0.961–0.989; the samples taken from LC and HC groups at 36 h were more divergent than 12 h (Pearson correlation coefficient was about 0.838–0.877 at 12 h and about 0.536–0.651 at 36 h). These results suggest that that the collected samples were reasonable with good correlations among the biological replicates. Comparative transcriptome data of HC with LC groups at 12 h groups showed 930 differentially expressed genes (DEGs) including 402 upregulated and 528 down-regulated genes. However, after 36 h of treatment, we could see that 1534 DEGs were significantly detected, including 768 up-regulated genes and 766 down-regulated genes. Venn diagram analysis showed that 521 genes were expressed significantly differently in both 12 h and 36 h intervals, including 218 up-regulated genes and 291 down-regulated genes (Figure 4A,B). 

### 2.3. Gene Ontology (GO) Enrichment and KEGG Pathway Analysis for DEGs

**Figure 5:**
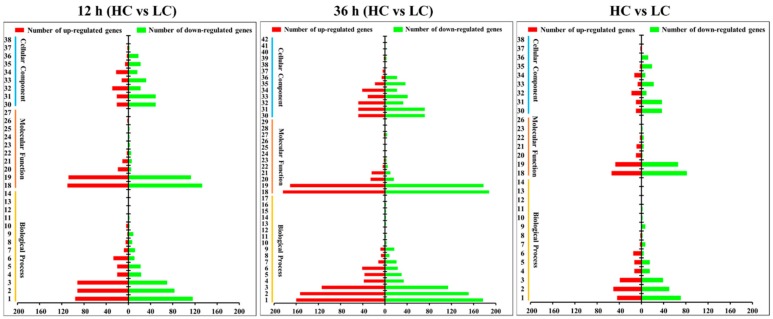
Gene ontology (GO) classification of DEGs between high (HC) and low (LC) CO_2_ treatments. Different numbers in the left of each bar chart represent the level 2 GO terms: 1. metabolic process; 2. cellular process; 3. single-organism process; 4. biological regulation; 5. regulation of biological process; 6. localization; 7. response to stimulus; 8. signaling; 9. cellular component organization or biogenesis; 10. positive regulation of biological process; 11. negative regulation of biological process; 12. detoxification; 13. multicellular organismal process; 14. developmental process; 15. reproduction; 16. reproductive process; 17. multi-organism process; 18. binding; 19. catalytic activity; 20. transporter activity; 21. nucleic acid binding transcription factor activity; 22. molecular function regulator; 23. antioxidant activity; 24. molecular transducer activity; 25. signal transducer activity; 26. nutrient reservoir activity; 27. structural molecule activity; 28. electron carrier activity; 29. transcription factor activity, protein binding; 30. cell; 31. cell part; 32. membrane; 33. organelle; 34. membrane part; 35. macromolecular complex; 36. organelle part; 37. extracellular region; 38. extracellular region part; 39. membrane-enclosed lumen; 40. virion; 41. virion part; 42. supramolecular fiber.

GO analysis showed that 878 DEGs (about 45.2% of total DEGs) gained GO annotation and were classified into three GO ontologies and 42 terms related to biological process, cellular components, and molecular functions (Figure 5 and Tables S3–S5). About 33 terms were enriched after 12 h of treatment, 42 terms were enriched after 36 h of treatment, and 30 terms were enriched at both time intervals. Most of the DEGs belonging to “Cellular Component” ontology showed down-regulated expression in the HC group at both 12 h and 36 h time intervals. The categories associated with the cell division process, including “cell” (GO:0005623), “cell part” (GO:0044464), “organelle” (GO:0043226), “macromolecular complex” (GO:0032991), “organelle part” (GO:0044422), and “cellular component organization or biogenesis” (GO:0071840) mainly contained down-regulated genes related to the HC treatment, suggesting a negative effect of a high CO_2_ concentration on pileus cell division. However, the GO categories of “localization” (GO:0051179), “transporter activity” (GO:0005215), and “nucleic acid binding transcription factor activity” (GO:0001071) chiefly contained up-regulated genes related to the HC treatment, which indicated the cell metabolism was still active under high CO_2_ conditions. The results also showed that the high CO_2_ treatment may down-regulate gene expression in the “response to stimulus” (GO:0050896) category.

**Figure 6:**
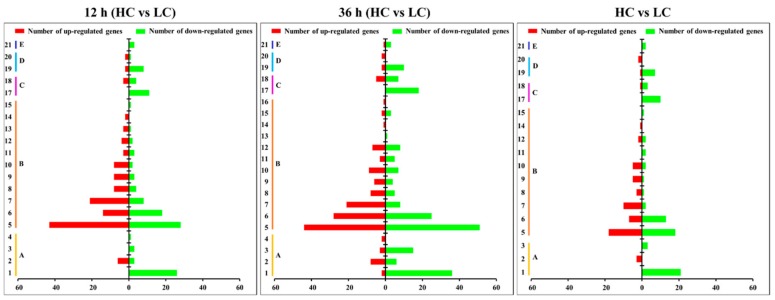
Kyoto Encyclopedia of Genes and Genomes (KEGG) classification of DEGs between high (HC) and low (LC) CO_2_ treatments. Different capital letters in the left of each bar chart represent the KEGG A Classes: A. Genetic Information Processing; B. Metabolism; C. Cellular Processes; D. Environmental Information Processing; E. Organismal Systems. Different numbers in the left of each bar chart represent the KEGG B Classes: 1. Folding, sorting and degradation; 2. Translation; 3. Replication and repair; 4. Transcription; 5. Global and overview maps; 6. Carbohydrate metabolism; 7. Amino acid metabolism; 8. Lipid metabolism; 9. Energy metabolism; 10. Nucleotide metabolism; 11. Metabolism of other amino acids; 12. Metabolism of cofactors and vitamins; 13. Metabolism of terpenoids and polyketides; 14. Biosynthesis of other secondary metabolites; 15. Glycan biosynthesis and metabolism; 16. Xenobiotics biodegradation and metabolism; 17. Cell growth and death; 18. Transport and catabolism; 19. Signal transduction; 20. Membrane transport; 21. Aging.

The Kyoto Encyclopedia of Genes and Genomes (KEGG) pathways of the DEGs are shown in Figure 6 and Tables S6–S8. The figure shows that 103 pathways, which can be classified into five groups and 21 subgroups, enriched 469 DEGs (about 24% of the total DEGs). The “Genetic Information Processing”, “Cellular Processes”, “Environmental Information Processing”, and “Organismal Systems” groups showed significantly higher numbers of down-regulated genes than up-regulated genes (only “translation” and “membrane” subgroups showed higher numbers of up-regulated genes). Especially, the subgroups of “Folding, sorting and degradation”, “Replication and repair”, “Cell growth and death”, and “Signal transduction” enriched more than 10 DEGs for each and almost exclusively contained down-regulated genes following both 12 h and 36 h treatments. The up-regulated genes were mainly enriched in the “Metabolism” group, especially in the “Global and overview maps”, “Carbohydrate metabolism”, and “Amino acid metabolism” subgroups. These findings correspond to GO annotation results and demonstrate that the metabolic pathways were active under high CO_2_ conditions.

### 2.4. Proteasome and Cell Cycle Pathways were Down-Regulated under High CO_2_ Conditions

**Figure 7:**
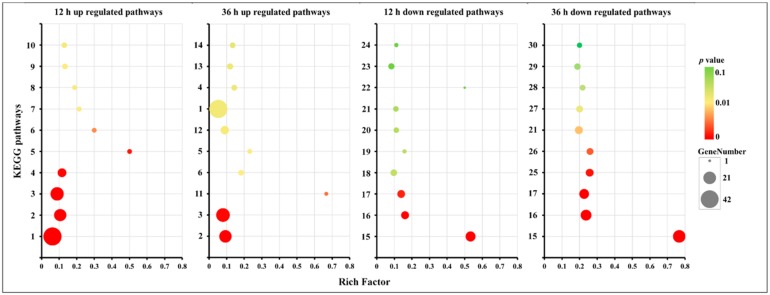
Bubble diagrams of the top 10 enriched KEGG pathways of the HC group compared with the HC group. Different numbers in Y-axis represent the KEGG pathways: 1. Metabolic pathways; 2. Biosynthesis of antibiotics; 3. Biosynthesis of secondary metabolites; 4. Biosynthesis of amino acids; 5. Fatty acid biosynthesis; 6. Valine, leucine and isoleucine biosynthesis; 7. Sulfur metabolism; 8. Terpenoid backbone biosynthesis; 9. 2-Oxocarboxylic acid metabolism; 10. Cysteine and methionine metabolism; 11. C5-Branched dibasic acid metabolism; 12. Fructose and mannose metabolism; 13. Glycine, serine and threonine metabolism; 14. Purine metabolism; 15. Proteasome; 16. Meiosis–yeast; 17. Cell cycle–yeast; 18. Protein processing in endoplasmic reticulum; 19. Phosphatidylinositol signaling system; 20. Starch and sucrose metabolism; 21. Amino sugar and nucleotide sugar metabolism; 22. Sesquiterpenoid and triterpenoid biosynthesis; 23. MAPK signaling pathway–yeast; 24. Longevity regulating pathway–multiple species; 25. DNA replication; 26. Citrate cycle (TCA cycle); 27. Glycolysis/Gluconeogenesis; 28. Base excision repair; 29. Nucleotide excision repair; 30. Homologous recombination. The rich factor represents the ratio between the DEGs and all annotated genes enriched in the pathway. The bubble scale represents the number of different genes, and the depth of the bubble color represents the adjusted *p*-value.

The top 10 enriched pathways (Figure 7 and Supplementary Table S9) demonstrated that the up-regulated DEGs were mainly enriched in the “Global and overview maps” subgroup, including four KEGG pathways: “metabolic pathways” (map01100), “Biosynthesis of antibiotics” (map01130), “Biosynthesis of secondary metabolites” (map01110), and “Biosynthesis of amino acids” (map01230). It was surprising that “proteasome” (map03050, belonging to the folding, sorting, and degradation subgroup) had 16 down-regulated genes that were detected following the 12 h treatment and 23 down-regulated genes that were detected following the 36 h treatment. The rich factor increased from 0.53 to 0.77 with the increase of treatment duration, which was the most significant enriched pathway of DEGs. Followed by, the “cell cycle–yeast” (map04111, belonging to the cell growth and death subgroup), which had 10 and 17 down-regulated genes mapped following the 12 h and 36 h treatments, respectively. The rich factor increased from 0.16 to 0.24. The “meiosis–yeast” (map04113) was also enriched in both the 12 h and 36 h down-regulated genes. However, the lamella (the region for meiosis and basidiospore production of the fruiting body) was completely removed from the pileus and only the context part was collected for RNA-seq. This means that the meiosis process could not take place in these samples. Further analysis found that all of the DEGs (except for g4645) mapped to the “meiosis–yeast” pathway also belonged to the “cell cycle–yeast” pathway. According to the bubble diagram, “proteasome” and “cell cycle–yeast” were the main pathways down-regulated under high CO_2_ conditions. 

**Figure 8:**
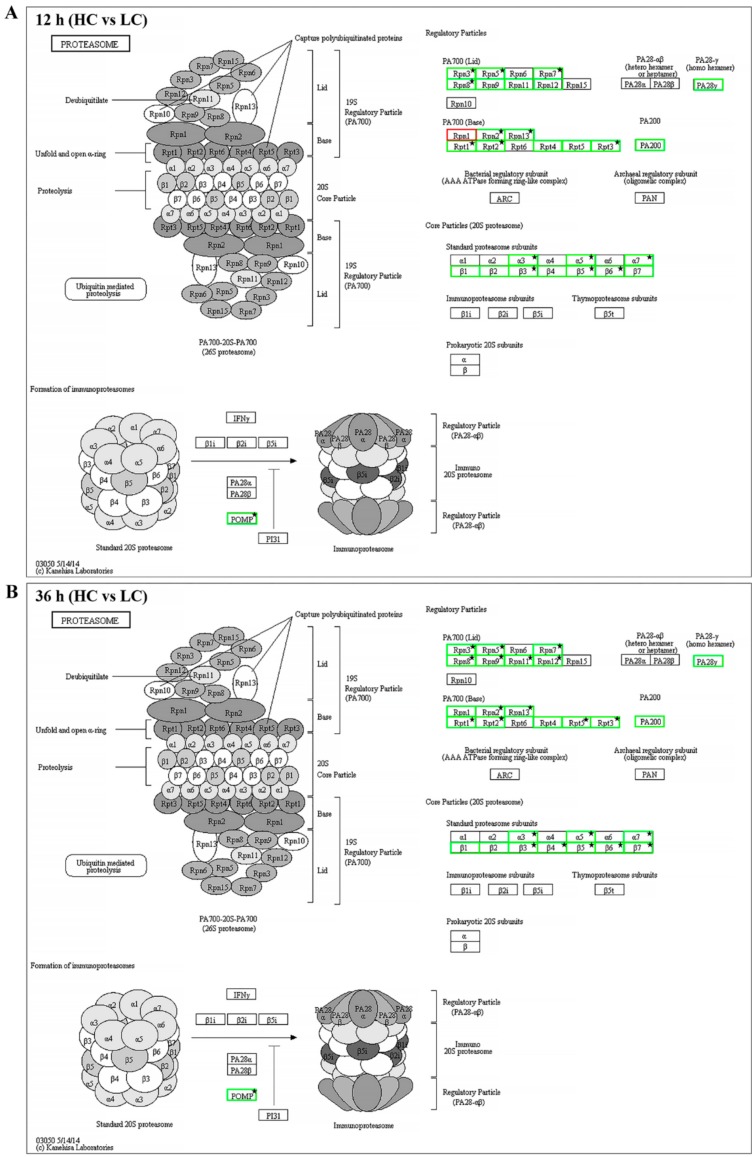
Putative pathway of proteasome in *F. filiformis* generated by KEGG analysis. (**A**) The gene expression profiles between high (HC) and low (LC) CO_2_ treatments at 12 h. The enzymes encoded by DEGs between HC and LC at 12 h are marked by stars. (**B**) The gene expression profiles between HC and LC at 36 h. The enzymes encoded by DEGs between HC and LC at 36 h are marked by stars. The boxes with a red border indicate up-regulation in the high CO_2_ treatment. The boxes with a green border represent down-regulation in the high CO_2_ treatment. The boxes with a black border mean no genes were annotated.

**Figure 9:**
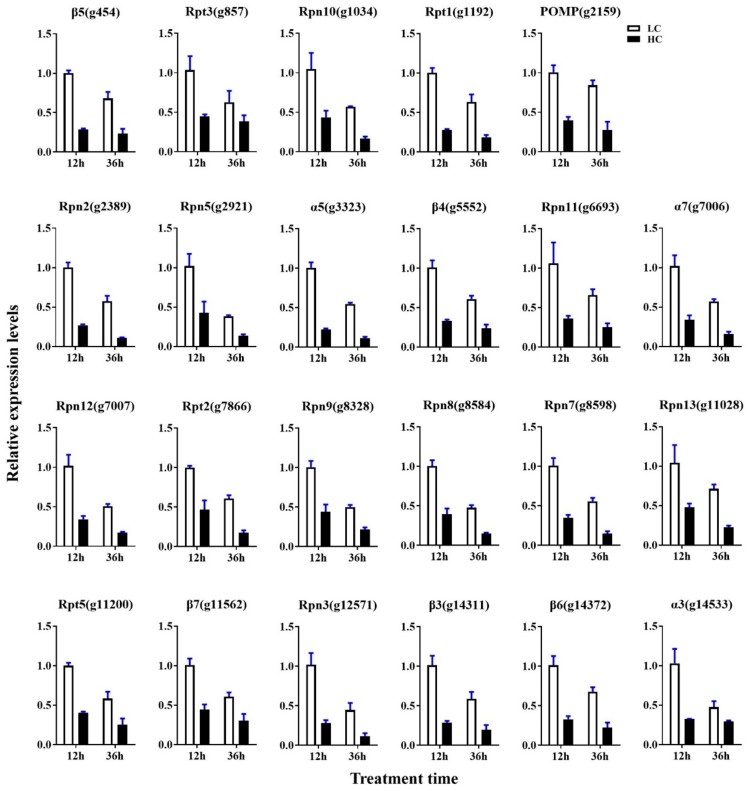
qRT-PCR analysis of DEGs in the proteasome pathway. Data are the means of three biological replicates, and the bars represent SEMs.

**Figure 10:**
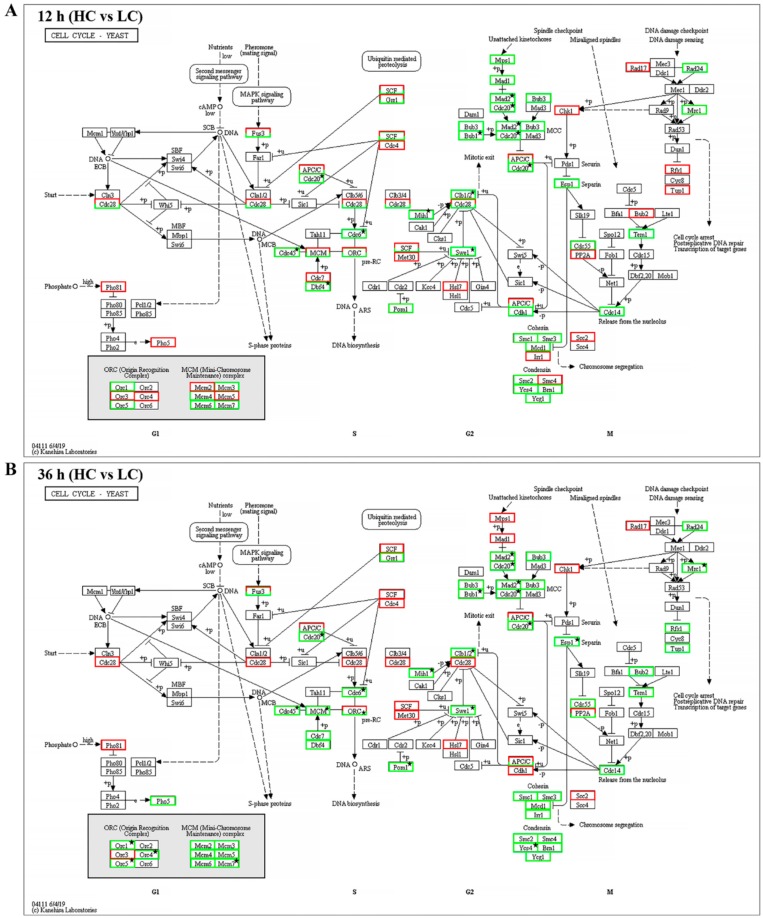
Putative pathway of cell cycle–yeast in *F. filiformis* generated by KEGG analysis. (**A**) The gene expression profiles between high (HC) and low (LC) CO_2_ treatments at 12 h. The enzymes encoded by DEGs between HC and LC at 12 h are marked by stars. (**B**) The gene expression profiles between HC and LC at 36 h. The enzymes encoded by DEGs between HC and LC at 36 h are marked by stars. The boxes with a red-border indicate up-regulation in the high CO_2_ treatment. The boxes with a green border represent down-regulation in the high CO_2_ treatment. The boxes with a black border mean no genes were annotated. The solid arrows indicate activation, the dashed arrows mean indirect effect.

**Figure 11:**
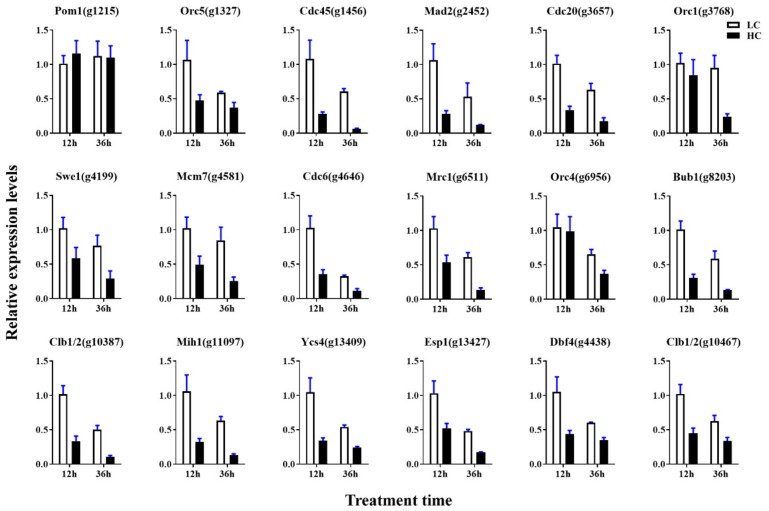
qRT-PCR analysis of DEGs in the proteasome pathway. Data are the means of three biological replicates, and the bars represent SEMs.

For further investigation of the proteasome and cell cycle processes under different concentrations of CO_2_, all expressed genes were mapped into these two pathways. As shown in Figure 8A,B and Supplementary Table S10, 30 genes were mapped to 29 enzymes in the proteasome pathway, 29 genes of 28 enzymes showed lower expression levels in the HC group with both 12 h and 36 h intervals (including 16 DEGs of 12 h and 23 DEGs of 36 h), and only Rpn1 (k03028) encoding the gene *g10463* showed higher expression (|log2 FC|= 0.06, FDR = 0.89) in the HC group after 12 h of treatment. The expression levels of all 23 DEGs were verified by qRT-PCR. The transcriptional levels of these genes in the LC groups were about 1.6–5.3-fold greater than in the HC groups, which was correlated with the transcriptome profiling data (Figure 9). As shown in Figure 10A,B and Supplementary Table S11, 58 enzymes of the cell cycle–yeast pathway were mapped by 72 expression genes (APC/C mapped six genes, SCF mapped four genes, Fus3 mapped three genes, and Bub1, Cdc28, Clb1/2, Grr1, PP2A, and Tup1 mapped two genes each). Among them, 10 DEGs were detected following 12 h of treatment and 17 DEGs were detected following 36 h of treatment. All of these DEGs were down-regulated in HC groups. The expression levels of all 18 DEGs were verified by qRT-PCR, and the transcriptional levels of these genes (except for g1215) in the LC groups were higher than in the HC groups, which was correlated with the transcriptome profiling data (Figure 11). The FPKM analysis showed that g1215 had low expression in all the samples and had the highest FPKM value of 1.3.

Interestingly, both the qRT-PCR and transcriptome profiling data show that the genes expression in the proteasome and cell cycle–yeast pathways decreased with increased incubation time under the same CO_2_ concentration (Figure 9 and Figure 11, Supplementary Tables S10 and S11). This phenomenon is more obvious in the low CO_2_ concentration group. As proteasome and cell cycle–yeast are two main pathways involved in cell division [32,33], cell division speed may become slower as the pileus grows bigger.

### 2.5. Ubiquitin-Conjugating Machinery Showed Differential Gene Expression under Different CO_2_ Conditions

**Figure 12:**
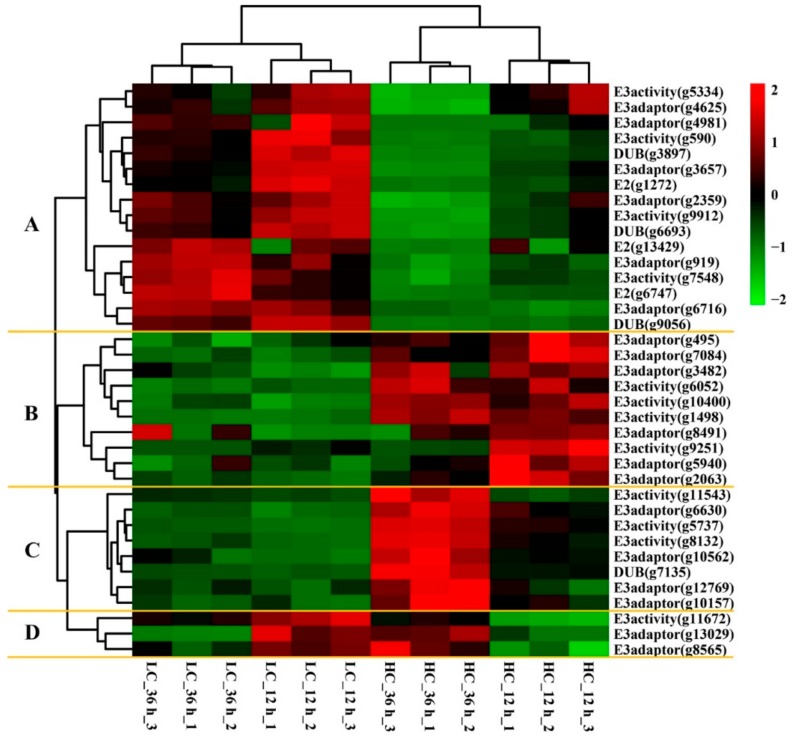
Heat map diagram showing the expression profiles of ubiquitin-conjugating related differentially expressed genes (DEGs). The right side of the heatmap indicates the enzyme names in the UUCD database and the gene ID of *F. filiformis*. Four main clades based on expression patterns were divided by yellow lines. The gene expression values (FPKMs) were transformed to Z-score values.

The ubiquitin–proteasome system is involved in the regulation of many basic cellular processes [32]. The covalent modification of proteins with ubiquitin is necessary for a tight cell cycle and division in all eukaryotes [34]. The KEGG mapping results demonstrate that a high CO_2_ concentration can down-regulate gene expression of both the proteasome and cell cycle–yeast pathways, so it is necessary to investigate the expression patterns of ubiquitin-related enzyme family genes. About 230 ubiquitin-related enzyme proteins of *Schizosaccharomyces pombe* were downloaded from the Ubiquitin and Ubiquitin-like Conjugation Database (UUCD) [35], and local blastp results showed that 37 ubiquitin relative enzyme encoding genes were annotated from 1943 DEGs, including three E2s (ubiquitin-conjugating enzymes or ubiquitin-carrier enzymes), 30 E3s (ubiquitin ligases), and four DUBs (deubiquitinating enzymes). The heat map of gene expression patterns showed that the DEGs were clustered into four main clades (Figure 12 and Supplementary Table S12). Clade A is the biggest clade, containing three E2s, three DUBs and 10 E3s that were up regulated in LC samples and down regulated in HC samples after 36 h. These results were consistent with the expression pattern of the proteasome and cell cycle–yeast pathway genes. Clade B contained 10 E3s with elevated expression in HC groups but minimum expression in LC groups. Clade C contained seven E3s and one DUB; these genes showed maximum expression in the 36 h HC samples, followed by the 12 h HC samples, while they showed low expression in the LC samples. There were only three E3 genes in clade D and they showed higher expression in the 12 h LC and 36 h HC samples. The LC and HC samples were clustered into two different clades, and the biological repeats of each treatment were clustered together into a single subclade. These results indicate that the gene expression of ubiquitin-conjugating machinery might be regulated by different CO_2_ concentrations.

## 3. Discussion

The pileus size is an important agronomic trait that determines the economic value of commercial mushrooms. Different mushroom species have different requirements for the pileus size from marketing and sales points of view. For example, large-sized pilei (but not senility) are preferred by consumers for *Lentinula edodes*, *Pleurotus ostreatus*, and *Clitocybe maxima*. However, smaller pilei are considered to have better quality and taste for *F. filiformis*, *P. eryngii*, and *Hypsizygus marmoreus*. In China, the pileus diameter has already become industrial standard of mushroom quality identification, and an automatic fresh mushroom sorting system based on pileus diameter has been developed [36]. Therefore, the production of mushrooms with appropriately sizes pilei has become a standard objective of all mushroom factories. Understanding the mechanism of pileus expansion is a basic requirement for a refined system to achieve a standard pileus size. However, the simultaneous developmental stages including expansion, basidiospore production, and ejection into the pileus make it hard to study the mechanism of its expansion alone. Until now, little has been known about the pileus expansion mechanism on a molecular level, and only a few genes related to pileus development have been identified in model species, i.e., *Coprinopsis cinerea* and *F. velutipes* [37,38]. In this study, the lamella was removed from pileus and only the context part of *F. filiformis* was collected for RNA-seq, which made it possible to explore the pileus expansion mechanism further.

For a long time, CO_2_ has been used to control fruiting-body morphogenesis during mushroom cultivation [39,40]. It has been reported that a high CO_2_ concentration can support thinning and elongation of the stipe and significantly inhibit pileus development and the rate of colony expansion in cultivation bottles in *F. velutipes* [2,5]. Although, the regulatory effect of CO_2_ on fruiting body development has been demonstrated in many studies, the mechanism is still unknown. The present study confirmed that a high CO_2_ concentration could significantly inhibit pileus expansion and basidiospore ejection of *F. filiformis*. In previous studies, it has also been well documented that the ubiquitin–proteasome pathway plays a critical role in the cell cycle process and leads to cell division [41,42]. The RNA-seq results suggest that CO_2_ has an inhibitory effect on the ubiquitin–proteasome and cell cycle–yeast pathways. These results support the idea that the high CO_2_ down-regulated ubiquitin–proteasome system decreases the rates of cell division, inhibiting pileus expansion.

Previously, it has been reported that the CO_2_ concentration showed no significant effect on the stipe length for fruiting bodies larger than 10 mm in *F. velutipes* [5]. The results from the present study also support the idea that a high CO_2_ concentration inhibits pileus expansion significantly but has a weak effect on stipe elongation. We also noticed that the stipe grows much faster than the pileus during the early stage of fruiting body development in *F. filiformis*. The fruiting-bodies used in our study had only a 2 mm diameter pileus, but the stipe length reached 50 mm. We suppose that the cell elongation, instead of cell division, became the main reason for stipe elongation in the fruiting bodies of this study. A high CO_2_ concentration may inhibit stipe cell division, but KEGG annotation results showed that a high CO_2_ concentration may up-regulate the gene expression of “Carbohydrate metabolism”, “Amino acid metabolism”, and some other “Metabolism” related pathways, which may positively regulate stipe cell elongation. Differences in growth rates between the stipe and pileus make it possible to produce the long stipe and small pileus fruiting bodies by controlling the CO_2_ concentration during *F. filiformis* cultivation.

**Figure 13:**
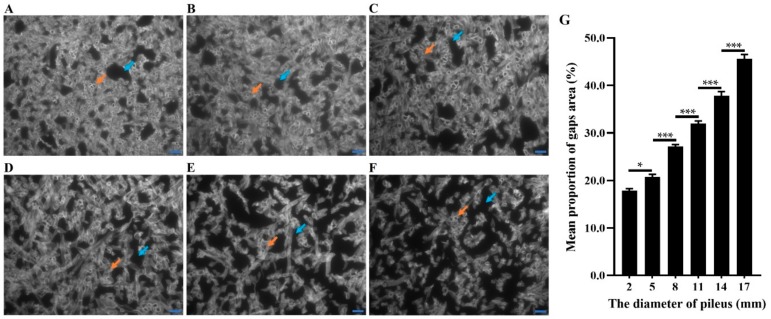
Observation of fluorescent whitening agent staining on paraffin sections of *F. filiformis* context cells from different diameters of the pileus: (**A**) 2 mm; (**B**) 5 mm; (**C**) 8 mm; (**D**) 11 mm; (**E**) 14 mm; and (**F**) 17 mm. The black areas indicated by the blue arrows are the gaps among hyphae. The white or gray areas indicated by the orange arrows are hyphae and hyphae transections. Bar = 20 μm. (**G**) The mean proportion of the gap area was quantified by image J software; data are the means of eight replicates, and the bars represent SEMs. Significant differences were analyzed using Tukey’s multiple comparisons test, * *p* < 0.05, *** *p* < 0.001.

Transcriptome and qRT-PCR data suggested that the gene expression in cell division pathways was down-regulated as the pileus grew bigger. However, a quick expansion in the pileus was also noted even after 36 h under LC treatment. This means that cell division is mainly involved in early pileus expansion, and there are some other reasons that may also promote the pileus becoming bigger. Anatomical observations showed that the context became spongy in over size pilei, especially, in the central region of the context (Figure 2). Morphological observations of the context tissues revealed that a few gaps appeared among the hyphae during the early pileus expansion stages (diameter of about 2 to 5 mm; Figure 13A,B). However, more and bigger gaps were observed as the pileus diameter increased from 5 to 17 mm (Figure 13B–F). The mean proportion of gaps area was significant increased from 17.9% to 45.6% during pileus expansion (from 2 to 17 mm in diameter; Figure 13G). The results indicate that the gaps between hyphae may play one of the main roles in promoting pileus expansion in pilei with a diameter of over 5 mm. These results combined with transcriptome data suggest that a high CO_2_ concentration could inhibit pileus expansion by decreasing the gene expression of the cell-division-related pathways during the early stage of fruiting body development.

In summary, this study systematically revealed the DEGs and significantly enriched GO terms and KEGG pathways between low and high CO_2_ concentrations using RNA-seq technology. We found that the ubiquitin–proteasome system and cell cycle–yeast pathways were the most significantly enriched with differentially expressed genes between low and high CO_2_ concentrations and might play roles in cell division in early pileus expansion. Our results also indicate that elevating the CO_2_ concentration during the early stage would lead to better inhibitory effects on pileus expansion. The results were consistent with the practical experience of mushroom cultivation farms and provide a theoretical basis for the carbon dioxide management strategy in *F. filiformis* and other types of mushroom cultivation. Moreover, our research found that the gaps in the context among hyphae might also be due to pileus expansion, especially in the middle and later pileus development stages. However, since the RNA-seq samples used in this research were limited to the early pileus expansion stages, the gap expansion mechanism is still unknown. In future studies, it would be worth investigating the mechanism of gap formation and expansion during pileus development, which may provide new ideas to develop a management strategy for pileus expansion rate control during mushroom cultivation. 

## 4. Materials and Methods 

### 4.1. Strain Maintenance and Fruiting Body Cultivation Methods

A commercial dikaryotic strain of *F. filiformis*, F19 (a hybrid that form a yellow fruiting body and was obtained by mating monokaryotic L11 and L22 strains), was obtained from the Fujian Edible Fungi Germplasm Resource Collection Center of China. The strain was maintained on potato dextrose agar medium (200 g/L potato; 20 g/L glucose; 20 g/L agar) at 25 °C. The cultivation of F19 strain fruiting bodies was carried out according to Tao et al. (2019) [43]. After the pileus differentiation stage, two sets of cultivation bottles (each set contained 12 bottles) were maintained separately at low (0.05%) and high (5%) CO_2_ concentrations.

### 4.2. Growth of Fruiting Bodies under Different CO_2_ Concentrations 

**Figure 14:**
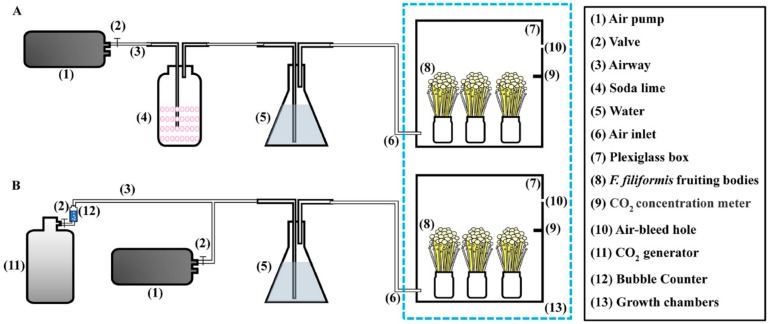
CO_2_ concentration controlling apparatus for the fruiting body cultivation of *F. filiformis*. (**A**) The cultivation apparatus with a low CO_2_ concentration. (**B**) The cultivation apparatus with a high CO_2_ concentration.

A special apparatus was used (Figure 14) to study the pileus growth of *F. filiformis* under different concentrations of CO_2_. Fruiting bodies of the *F. filiformis* F19 strain at a diameter of 2 mm at the pileus stage were moved into two 40 cm × 40 cm × 40 cm plexiglass boxes, and the boxes were sealed by plexiglass covers. The air and CO_2_ pumps were used to maintain a high concentration of CO_2_, while the soda lime was used to react with air CO_2_ to keep the CO_2_ at a low level in separate boxes. A specific CO_2_ concentration measuring meter (Vaisala, Finland) was used to monitor the CO_2_ concentration in the box. The gas was passed through aqueous solution to maintain a high humidity (about 90%) in the growth boxes. Boxes with high and low CO_2_ concentrations were put into the same growth chamber, and the temperature was adjusted to 10–12 °C. The low concentration (LC) of carbon dioxide was set as 0.05%, and the high concentration (HC) of carbon dioxide was set as 5%. 

### 4.3. Comparison of Fruiting Body Morphology, Pileus Size, and Basidiospore Ejection among Different CO_2_ Concentration

Fruiting bodies of strain F19 were grown under two different CO_2_ concentrations (0.05% and 5%) for 72 h. The morphology of fruiting bodies, pileus size, and basidiospore ejection were recorded every 12 h. For size measurement, the pileus was separated from the stipe and the diameter of it was measured using Vernier calipers (Meinaite, Shanghai, China), To study the effect of LC and HC of CO_2_ on basidiospore ejection, separated pilei were placed with the lamellas downwards onto the black paper for 12 h to eject basidiospores. Six replicates were included for each treatment. 

### 4.4. Sample Collection

The pileus is mainly composed of two parts: the context and the lamella (Figure 2). The context is the main part for pileus expansion, while the lamella is a place for meiosis and basidiospore generation and ejection [44]. To study the pileus expansion mechanism, the context part of the pileus was separated from the fruiting body by scalpel under an integrated type microscope SMZ168 (MOTIC, Guangzhou, China) and then immediately frozen in liquid nitrogen for RNA isolation. Each treatment was repeated three times and each sample contained 12 contexts. 

### 4.5. RNA Extraction, Library Construction, and Sequencing

Total RNA was isolated from frozen samples using an E.Z.N.A.™ Plant RNA Kit (Omega, Stamford, CT, USA) according to the manufacturer’s protocol. The quality of the extracted RNA was assessed on 1% agarose gel. The RNA purity, integrity, and concentration were also evaluated using a nanophotometer spectrophotometer (Implen, Westlake Village, CA, USA) and RNA Nano 6000 assay kit of the Agilent Bioanalyzer 2100 system (Agilent Technologies, Santa Clara, CA, USA). The RNA libraries were prepared using a NEB Next Ultra RNA Library Prep Kit for Illumina (NEB, Ipswich, MA, USA) following the manufacturer’s recommendations. The complementary DNA (cDNA) libraries were sequenced on an Illumina Novaseq platform (Illumina Inc., San Diego, CA, USA) at Novogene Co. Ltd (Tianjin, China), and 150 bp paired-end reads were generated. 

### 4.6. Sequence Read Mapping to the F. filiformis Reference Genome

Raw data (raw reads) in fastq format were first processed to remove adapters, and paired reads were removed if one of them contained more than 10% poly-Ns or more than 50% low-quality (Q ≤ 5) base sequences. Obtained clean reads were aligned to the reference genome (Bioproject ID 191865) of *F. filiformis* strain L11 [45] using HISAT2 with default parameters [46]. 

### 4.7. Quantification and Differential Expression Analysis of Transcripts

Differential expression analysis of the four groups (three biological replicates per condition) was performed using StringTie v.1.3.3 with default parameters [47]. The Ballgown v.2.10.0 R package [48,49] was used to analyze the differentially expressed genes (DEGs). Fragments per kilobase of transcript per million fragments mapped (FPKM) values served as measurement units to estimate the expression level of each gene. The samples’ relationships were analyzed by the Pearson correlation coefficient. A false discovery rate (FDR) <0.05 and |log2 (fold-change)| >1 were set as the thresholds for differentially expressed genes (DEGs) identification. GO annotation was done using Blast2GO v.5.2 [50]. The amino acid sequences were submitted to the KEGG Automatic Annotation Server (KAAS) for KEGG annotation [51]. The heat map and Venn diagram drawing and GO and KEGG enrichment analyses of DEGs were carried out using OmicShare (www.omicshare.com/tools) and BMKCloud (http://www.biocloud.net/). Euclidean distance and K-means algorithms were used to generate the heatmaps, significantly enriched GO terms and KEGG pathways in DEGs compared to the genome background were defined by hypergeometric tests. The local blastp program (e-value < 1 × 10^−5^) and NCBI Conserved Domains database (https://www.ncbi.nlm.nih.gov/cdd/) were used for homolog gene identification. The raw Illumina sequencing data were deposited in NCBI under Bioproject ID PRJNA557581.

### 4.8. Validation of the DEGs by qRT-PCR

The first strand cDNA was synthesized, and qRT-PCR was performed according to our previously reported methods [52]. Relative gene expression levels were calculated using the 2^−ΔΔ*C*t^ method [53]. The housekeeping genes of Glyceraldehyde-3-dehydrogenase (*gapdh*), β -actin (*actb*), and the new internal control gene of Ras-related small GTPase (*ras*) were used as reference genes [54]. The primers used for quantification in the study were designed using NCBI Primer-BLAST (https://www.ncbi.nlm.nih.gov/tools/primer-blast/). The qRT-PCR primer sequences can be seen in Table S13 and the data obtained represent three biological replicates.

### 4.9. Preparation of Paraffin-Embedded Context Tissue for Morphology 

Small thin sections were cut from the central region of the context in the pilei (Figure 2) using a fine dissection blade and fixed with FAA (formaldehyde–acetic acid–alcohol–water (2:1:10:7)) fixing solution for 12 h at 4 °C. The well-fixed samples were washed and dehydrated in a series of ethanol solutions, i.e., 20%, 30%, 40%, 50%, 60%, 70%, 75%, 80%, 85%, 90%, and 95% (about 30 min at each concentration), and finally, they were placed in anhydrous ethanol for 90 min. Later, dehydrated samples were treated with xylene–anhydrous ethanol solutions (1:3, 1:1, 3:1, and 1:0 for 60 min in each solution) to remove the ethanol. Finally, the samples were embedded and made into paraffin [55]. Well prepared paraffin sections were placed in a drop of Calcofluor White stain (Sigma, Saint Louis, MO, USA) for 1 min, placed under a cover slip, and observed using fluorescence microscopy BX51 (Olympus, Kyoto, Japan). Image processing software (Image J version 1.51J8) was used to quantify the mean proportion of the gap area.

### 4.10. Statistical Analysis 

In this study, the estimated standard error of the mean (SEM) was used to present the characteristics of the sample data. The significance tests were performed using Student’s *t*-tests or Tukey’s multiple comparisons tests with GraphPad Prism 6.01 software [56]. 

## Supplementary Materials

Supplementary materials can be found at https://www.mdpi.com/1422-0067/20/23/5923/s1. Table S1. Summary of transcriptome sequencing. Table S2. The FPKM values of 10726 genes. Table S3. GO functional classification of DEGs between HC and LC treatments at 12 h. Table S4. GO functional classification of DEGs between HC and LC treatments at 36 h. Table S5. GO functional classification of DEGs between HC and LC treatments with the same expression patterns in both 12 h and 36 h treatments. Table S6. KEGG functional classification of DEGs between HC and LC treatments at 12 h. Table S7. KEGG functional classification of DEGs between HC and LC treatments at 36 h. Table S8. KEGG functional classification of DEGs between HC and LC treatments with similar expression patterns in both 12 h and 36 h treatments. Table S9. The top 10 significantly gene enriched KEGG pathways of the HC treatment compared to the LC treatment. Table S10. The FPKM values of genes involved in the putative proteasome pathway in *F. filiformis*. Table S11. The FPKM values of genes involved in the putative cell cycle–yeast pathway in *F. filiformis*. Table S12. The FPKM values of DEGs involved in the ubiquitin-conjugating system in *F. filiformis*. Table S13. Sequences of primers used for qRT-PCR assays.

## Abbreviations

cDNA	complementary DNA
CO_2_	Carbon dioxide
DEGs	Differentially expressed genes
FC	Fold change
FDR	False discovery rate
FPKM	Fragments per Kilobase Million
GO	Gene ontology
HC	High concentration
KEGG	Kyoto Encyclopedia of Genes and Genomes
LC	Low concentration
qRT-PCR	Quantitative Real-time PCR
